# Unravelling the Roles of Susceptibility Loci for Autoimmune Diseases in the Post-GWAS Era

**DOI:** 10.3390/genes9080377

**Published:** 2018-07-27

**Authors:** Jody Ye, Kathleen M. Gillespie, Santiago Rodriguez

**Affiliations:** 1 Diabetes and Metabolism, Translational Health Sciences, University of Bristol, Level 2 Learning and Research Building, Southmead Hospital, Bristol BS10 5NB, UK; k.m.gillespie@bristol.ac.uk; 2 MRC Integrative Epidemiology Unit, Population Health Sciences, University of Bristol, Bristol BS8 2BN, UK; santi.rodriguez@bristol.ac.uk

**Keywords:** complex loci, autoimmune diseases

## Abstract

Although genome-wide association studies (GWAS) have identified several hundred loci associated with autoimmune diseases, their mechanistic insights are still poorly understood. The human genome is more complex than single nucleotide polymorphisms (SNPs) that are interrogated by GWAS arrays. Apart from SNPs, it also comprises genetic variations such as insertions-deletions, copy number variations, and somatic mosaicism. Although previous studies suggest that common copy number variations do not play a major role in autoimmune disease risk, it is possible that certain rare genetic variations with large effect sizes are relevant to autoimmunity. In addition, other layers of regulations such as gene-gene interactions, epigenetic-determinants, gene and environmental interactions also contribute to the heritability of autoimmune diseases. This review focuses on discussing why studying these elements may allow us to gain a more comprehensive understanding of the aetiology of complex autoimmune traits.

## 1. Introduction

Autoimmune disease is a major human health burden, affecting 5% to 8% of the world’s population. To date, more than 80 autoimmune diseases have been described [1]. For some of the more common conditions, such as multiple sclerosis, Crohn’s disease, rheumatoid arthritis, and type 1 diabetes, increases in incidence and prevalence have been observed in westernised societies over the last several decades [2,3,4,5]. Both genetic and environmental factors are thought to play roles in the initiations and progression of autoimmunity. Environmental factors such as viral infection [6], nutrition [7], gut dysbiosis [8] and in utero environment [9] have all been postulated to play a role but have been difficult to confirm due to variations among individuals, populations and geographical areas [10,11,12,13,14,15]. In contrast, common genetic components of autoimmune diseases have been better characterised.

In the 1970s, studies of twins and first-degree relatives identified the most important genetic risk attributed by the human leukocyte antigen (HLA) region [16,17,18,19,20]. In the subsequent decades, candidate gene studies led to the discovery of several other predisposing loci for autoimmune diseases. For example, variable number tandem repeat (VNTR) class I alleles upstream of the insulin gene were associated with type 1 diabetes [21,22,23]; *PTPN22* was found to be associated with rheumatoid arthritis, type 1 diabetes, systemic lupus erythematosus, and Grave’s disease [24]; *CTLA4* was associated with a number of autoimmune conditions including autoimmune thyroid disease, type 1 diabetes, coeliac disease [25]; and *IL2RA* was associated with type 1 diabetes, rheumatoid arthritis, and multiple sclerosis [2,26,27]. The subsequent genome-wide association studies (GWAS) comparing affected cases with unrelated healthy individuals substantially increased the power of gene discovery analyses for autoimmune diseases, which led to over 300 loci being identified [28]. GWAS improved our knowledge of disease risk, but the specific design of GWAS does not allow consideration of other elements potentially involved in disease susceptibility. This is because GWAS are based on common genetic variants, specifically single nucleotide polymorphisms (SNPs). While the aetiologies of ‘complex diseases’ are attributed by genetic and environmental components, as well as the interplay between the both, genetic factors themselves are comprised of SNPs and genetic variations at many other levels. In addition, phenomena such as gene-gene interactions (epistasis), and genetic-epigenetic interactions increase the complexity of the genetic basis of human common diseases and traits. Recent studies suggest that many causal variants trigger autoimmune responses in a cell-type specific and/or cell-state specific manner. In this review, we discuss the roles of complex loci at genetic and epigenetic levels in the aetiology of autoimmune diseases.

## 2. The Genome-Wide Association Studies Era

Genome-wide Association Studies were facilitated by advancing technology to conduct high-throughput SNP analyses in very large case-control populations. For autoimmune diseases, by identifying susceptible genetic variants, one can (a) help understand the underlying biological pathways to inform the design of novel immune therapies; and (b) predict the risk of individuals developing autoimmunity. One of the strengths of GWAS is its study design. The powerful hypothesis-free, association mapping design of GWAS has enabled the identification of hundreds of candidate genes strongly associated with human traits. Briefly, a GWAS is performed first by genotyping a set of SNPs (often over 500 k SNPs) using commercial microarrays. Subsequently, genotypes at the genome-wide level are imputed according to the haplotype structures provided by reference panels. For example, based on the early HapMap phase 1–3 reference panel, the Wellcome Trust Case Control Consortium published a number of GWAS for common autoimmune diseases such as rheumatoid arthritis and type 1 diabetes [29]. The 2.5 million SNPs from the HapMap reference panel only included the most common variants, with minor allele frequencies greater than 5% of the study population [30]. The subsequent 1000 Genomes Project interrogated genotypes of nearly 40 million variants, including 1.4 million insertions/deletion variants [31], which dramatically improved GWAS coverage, allowing the identification of several novel autoimmune disease loci [32]. One of the applications of GWAS results is the development of genetic risk score (GRS). Genetic risk score does not aim to detect individual SNPs, but instead is an aggregate of genetic risk across the genome. It is calculated by combining the effect sizes of multiple SNPs, weighted by the strength of each SNP [33]. Although GRS is not powerful enough to make clinical diagnosis at individual level [34], it has been shown to be useful for patient stratification and risk prediction [35,36,37,38]. For example, in type 1 diabetes, it was suggested that targeting the top 18% individuals of the general population with the highest ranked GRS would capture 80% of future cases [39]. Given that the prevalence of type 1 diabetes is 0.4% in the general population, this will decrease the number of individuals that will need to be treated to prevent one type 1 diabetes case from 250 to 50 [39].

## 3. Missing Heritability in Genome-Wide Association Studies

Narrow sense heritability (h^2^) is a term that defines the proportion of phenotypic variance that is contributed by additive genetic variance [40]. It has been shown that GWAS tend to explain a smaller fraction of h^2^ compared to those estimated from classical methods based on population data, such as from the analysis of offspring and parental phenotypes, siblings or monozygotic and dizygotic twins [41]. This gap is known as missing heritability [41]. Even in highly heritable conditions such as type 1 diabetes and juvenile idiopathic arthritis (h^2^ up to 90%), SNP based h^2^ still falls short of those reported from population estimates [41]. Missing heritability was originally thought to be partly contributed by rare variants of the genome. To examine this hypothesis, Hunt et al. first performed dense exon sequencing of 20 GWAS loci that exhibit shared susceptibilities to six common autoimmune diseases to identify putative rare variants and then conducted single-variant association analyses among the 20 loci; their findings suggested that rare variants have a negligible impact on autoimmune disease risk [42]. Thus, the components of missing heritability remain largely unknown and may involve complex loci, gene-gene interactions and epigenetic regulations [43,44].

## 4. The Role of Complex Loci at Genetic Level

Complex loci include many types of genetic variations, such as insertion-deletion polymorphisms (indels), VNTRs (including microsatellites and minisatellites), copy number variants (CNVs, with each DNA fragment that is ~1 kb or larger) [45], long interspersed nuclear elements [46], short interspersed nuclear elements, Alu repeats, somatic mosaicism, and cytogenetic abnormalities (including insertions, duplications, translocations, inversions). There is evidence demonstrating functional effects on human diseases caused by complex genetic variations of the genome [46,47]. One particular example is the VNTR region upstream of the *INS* promoter, as it was one of the very first loci to be associated with type 1 diabetes [48]. Of the three subclasses of *INS* VNTR, class I homozygous alleles were predisposing (odds ratio (OR) = 2.68) and class III alleles were dominantly protective (OR = 0.37) [21]. The predisposing class I variants were associated with lower levels of *INS* mRNA expression in the thymus, implying impaired tolerance induction of T-cells during thymic education [49]. Another example is the VNTR of the *C4* (complement component 4), which has been associated with systemic lupus erythematosus. Systemic lupus erythematosus is an autoimmune condition where deficiency in the clearance of apoptotic cells and immune complexes is involved as part of the disease aetiology [50]. The protein product of *C4* (C4A) plays a pivotal role in the activation of the classical and the lectin complement pathways that lead to cytolysis or neutralisation of invading microbes, clearance of immune complexes and apoptotic materials [51]. Low copy number of *C4* VNTR was strongly associated with increased risk of systemic lupus erythematosus and was coupled with lower levels of plasma C4A, whereas high copy number of *C4* was protective and was associated with higher levels of plasma C4A [51].

Due to the complexity of structural DNA elements, technical challenges exist to study them directly in large-scale populations. A simple way to profile their associations in common diseases is through tagged SNPs; for example, a given SNP in linkage disequilibrium with another genetic variant, such as a VNTR or CNV, could capture the genetic variations and therefore be used as a proxy. However, it is possible that not all of the structural elements are well-tagged by SNP arrays used to conduct GWAS, and hence, they are not directly tested in GWAS for their associations with autoimmune diseases. Sequencing studies showed that CNVs account for a major proportion of human genetic polymorphism [52,53]. To assess whether CNVs can explain missing heritability of common diseases, the Wellcome Trust Case Control Consortium employed custom arrays that interrogate approximately 50% of all common CNVs (>5% frequency) and performed association analyses for eight diseases, including bipolar disorder, breast cancer, coronary artery disease, Crohn’s disease, hypertension, rheumatoid arthritis, type 1 diabetes and type 2 diabetes; over 3400 polymorphic CNVs were surveyed [54]. Although the study identified three associated CNV loci—*HLA* for Crohn’s disease, rheumatoid arthritis and type 1 diabetes; *IRGM* for Crohn’s disease; and *TSPAN8* for type 2 diabetes, all of which were tagged by SNPs from previous GWAS [54]. However, a limitation of this study was that CNVs that were poorly assigned into distinct classes were eliminated from the analyses. A subsequent study by Zanda et al. further tested 3410 previously unclustered and untagged CNVs on their associations with type 1 diabetes using a family study design [55]. The authors observed no convincing associations between untagged CNVs and type 1 diabetes. Therefore, data from both studies led to the conclusion that common CNVs do not explain a major proportion of missing heritability. If CNVs are involved in susceptibility to common autoimmune diseases, one would imagine that they must be rare variants with large effect sizes. In fact, only 22% of rare CNVs (<1% frequency) were tagged by at least one SNP, indicating that they would be interesting targets for investigation [54]. Cooper et al. investigated whether rare CNVs could have a significant contribution to type 1 diabetes risk [56]. In their study, 6808 type 1 diabetes and 9954 control individuals were compared; de novo rare CNV deletions (rDELs) were found more frequently in type 1 diabetes patients. In addition, rare CNVs were divided into small (0–20 kb), medium (20–400 kb), and large (>400 kb) groups; there was a greater proportion of large rDELs in type 1 diabetes patients (OR = 1.57). More interestingly, very long CNVs were found in eleven type 1 diabetes cases and only one control (rDELs > 3 Mb, OR = 15.60). These rDELs are enriched in genes that regulate immune function. Because of the rarity of CNVs, the study had insufficient power and coverage. Nevertheless, these data still imply that rare CNVs are potentially involved in the risk for type 1 diabetes, but it was estimated that over 30,000 cases and equivalent number of controls are required to detect rare CNVs with large effects (ORs approximately 8) [56].

Another phenomenon that potentially contributes to missing heritability of autoimmune disease is somatic mosaicism. Unlike being passed from parents or developed ‘de novo’ in sperm or egg, this type of mutation occurs post-zygotically after the single cell stage, resulting in two genetically distinct cell populations within an individual [57]. Somatic mosaicism can be found in monozygotic twins; particularly, it was demonstrated in twins with neurological disorders [58], implying that it may play a role in disease pathogenesis. Somatic mosaicism has also been described in a number of autoimmune diseases. For example, de novo CNVs were previously found in the affected twins with primary biliary cirrhosis of monozygotic twin pairs [59]. Another example is autoimmune lymphoproliferative syndrome (ALPS), which is mainly characterised by the proliferation of CD4-/CD8- double negative lymphocytes [60]. These double negative T cells are originated from activated single positive T cells that had heterozygous somatic mutations of *Fas*, resulting in their resistance to apoptosis [61]. More rarely, somatic mosaicism has been reported to associate with a combination of autoimmune diseases. For example, a *KRAS* mutation was detected in T and B lymphocytes but not in natural killer cells of a patient with multiple autoimmunity, including thrombocytopenia, recurrent Henoch-Schonlein purpura and intestinal Behçet disease [62]. Further analyses are required to determine whether rare genetic variations such as rDELs and somatic mosaicism have significant additional value to SNPs in missing heritability of autoimmune diseases or if their effects are negligible. Other possible factors explaining missing heritability may include inaccurate heritability estimates, study design limitations of GWAS (such as sample size, type of genetic variants considered), non-additive genetic effects (epistasis, gene and environment interaction), epigenetics and disease heterogeneity [43,63,64].

Genome-wide Association Studies are set out to detect the most associated signals within a given genomic region. Interestingly, some regions such as the HLA locus demonstrate multiple association signals with autoimmune diseases. To better define independent association signals, conditional stepwise regression is often used. This was designed to ask the question whether a single variant could best explain a trait and conditional on this single variant, whether any other variants explain additional trait variance [65]. The international multiple histocompatibility complex and autoimmunity genetics network performed conditional regression analyses on seven autoimmune disorders in the HLA region [66]. For example, in systemic lupus erythematosus, the top associated signal was rs1269852, a SNP located between *TNXB* and *CREBL1* in chromosome 6, in strong linkage disequilibrium with HLA-DRB1*0301. Conditioning on the top variant identified multiple secondary association signals such as HLA-DRB1*1501, class I (between *RNF39* and *TRIM31*), class II (*HLA-DQB1-DQA2*), and class III (*NOTCH4*) HLA alleles. For Crohn’s disease, conditioning on the top signal HLA-DRB1*1101 identified additional independent associations such as rs382259 (located near the *NOTCH4* region), the class I region (between *HLA-B* and *MICA*) and the *DQA-DQB1-DQA2-DQB2* region. For multiple sclerosis, conditional analysis revealed that apart from the top association DRB1*1501 (tagged by rs3135391), HLA-B*4402 and HLA-C*0501 also appeared to have independent associations. Together, these data demonstrate that the HLA region exhibits complex and multi-locus effect for autoimmune associations.

## 5. Identifying Candidate Causal Variants Using Fine Mapping

Genome-wide Association Studies signals highlight regions of associations, but the lead variants may not be causal to disease phenotype. Identifying causal variants in regions associated with autoimmune diseases is a challenging task, often due to high linkage disequilibrium structure and multiple disease-causing variants in relatively close proximity. Therefore, various fine-mapping strategies have been developed and we chose *IL2RA* as an example to illustrate how fine-mapping strategies have helped to define candidate causal variants for several autoimmune diseases. The *IL2RA* (CD25) is located on chromosome 10p15.1; it encodes a subunit (IL-2Rα) of the receptor for the pro-inflammatory cytokine IL-2, which has been associated with a number of autoimmune diseases including multiple sclerosis, rheumatoid arthritis, autoimmune thyroid disease, and type 1 diabetes [2,27,67]. Upon stimulation of IL-2, IL-2 receptor signals to maintain the suppressive functions of CD4+FOXP3+ regulatory T cells and facilitate effector and memory T cell differentiation [68]. The *IL2RA* region was initially found to be associated with type 1 diabetes using a multi-locus genetic association test in 2005 [69]. Using conditional logistic regression as mentioned previously, Lowe et al. fine-mapped the region covering *IL2RA* and its neighbouring gene *RBM17*, which led to the identification of two loci independently associated with type 1 diabetes [70], each comprising a number of indistinguishable SNPs. Group 1 is located in intron1 of *IL2RA* (marked by rs12722495, previously marked by rs41295061) and Group 2 is located at the intergenic region between the 5′ of *IL2RA* and *RBM17* (marked by rs11594656) [70]. Maier et al. later discovered an independent Group 3 signal in intron 1 of *IL2RA*, tagged by SNP rs2104286 [71]. Apart from type 1 diabetes, Group 3 is also associated with multiple sclerosis. Functionally, the protective variant of the Group 1 SNP rs12722495 induces higher expression levels of CD25 on the surface of CD4+ memory T cells, potentially causing increased T cell activation in response to IL-2 stimulation. The protective variant of Group 3 SNP rs2104286 was counterintuitively associated with a lower percentage of CD25+ cells in CD4+ naïve fraction, suggesting a reduced likelihood of T cell activation [72].

**Figure 1:**
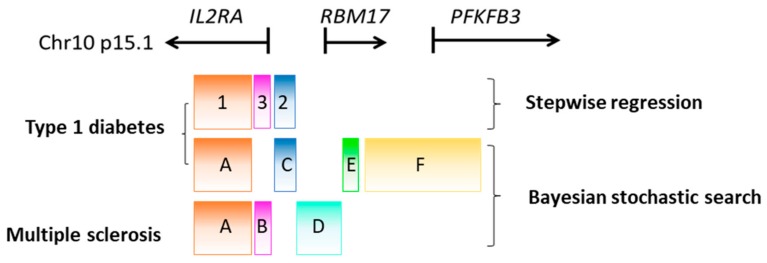
Schematic representation of multiple independent associations at the *IL2RA-RBM17-PFKFB3* region discovered using stepwise regression and Bayesian stochastic search that are associated with both type 1 diabetes and multiple sclerosis. Group 1–3 single nucleotide polymorphisms that contribute to type 1 diabetes were originally identified using stepwise regression. The later-developed Bayesian stochastic search, however, identified four groups of SNPs (A, C, E, F) that jointly explain type 1 diabetes risk at the *IL2RA* locus; whereas for multiple sclerosis, the risk at this locus was explained by Group A and D jointly, or by Group B SNPs alone. Figure was adapted and modified from [65].

The recent development of fine-mapping methods such as Bayesian stochastic search proved to be more efficient in detecting multiple independent association signals. Unlike conditional logistic regression, Bayesian stochastic search tests the question which sets of SNPs can best jointly explain type 1 diabetes association. Wallace et al. applied this approach to re-analyse the *IL2RA* region in 2015. They showed that, instead of three, there are four groups of SNPs independently associated with type 1 diabetes [65], with Group A SNPs located in the intron 1 of *IL2RA* (that is equivalent to Group 1), Group C SNPs located in the intergenic region between *IL2RA* and *RBM17* (equivalent to Group 2), which replaced the previous Group 3 SNP, Group E SNPs located at the 5′ of *RBM17*, and Group F SNPs resided in the 5′ of *RBM17* to intron 2 of *PFKFB3* (Figure 1) [65]. For multiple sclerosis, the risk could be explained either by Group A and Group D SNPs (tagged by rs56382813) jointly or by Group B SNP (rs2104286) alone.

## 6. Epistasis in Autoimmune Diseases

Genes may not function in isolation. Gene–gene interactions (epistases) are major contributors to autoimmune disease risk; a classical example is the HLA class II haplotypes. For example, HLA-DRB1*1501-DQB1*0602 is the most susceptible haplotype for multiple sclerosis [73] and HLA-DRB1*0301–DQB1*0201 is the most susceptible haplotype for type 1 diabetes [74]. A summary of HLA class II haplotypes in autoimmune diseases is reviewed here [75]. Class II and class I interactions also play a role in autoimmune disease risk. For instance, in type 1 diabetes patients, a combination of HLA-A*24, DQA1*03, and *DR9* has been associated with accelerated beta cell loss [76]. The interaction between *HLA-DR3/DR4* and class I A*03 allele demonstrated significant protective effect of clinical progression to type 1 diabetes, whereas *HLA-DR3/DR4* and class I B*39 interaction contributed significantly to the progression from multiple islet autoantibody to type 1 diabetes [77]. HLA also interact with non-HLA genes. For example, an increased risk of systemic lupus erythematosus (OR = 1.19) was observed when *CTLA4* (cytotoxic T lymphocyte antigen 4), a negative regulator of T cell response interacts with SNPs rs3131379 and rs1270942 located in the HLA class III region [78]. Another similar example was reported in multiple sclerosis. A driver of increased risk in multiple sclerosis is the soluble form of interleukin-7 receptor (IL-7R, encoded by *IL7R*). Exon 6 of *IL7R* interacts with many proteins, one of them is encoded by the *DDX39B* that is located in the HLA region. Galarza-Munoz et al. recently identified that a SNP rs2523506 within the *DDX39B* region reduces *DDX39B* expression. More importantly, the authors showed a significant increased risk in multiple sclerosis when rs2523506 interacts with a risk variant of *IL7R* (rs6897932). Carriers of risk alleles of both SNPs increased soluble IL-7R expression, thereby increasing the disease risk [79]. Although epistasis at the HLA region is readily detectable because HLA genes are the main susceptibility genes for autoimmune diseases, interactions between HLA and non-HLA genes, as well as between two non-HLA genes are difficult to observe due to the limited power at the current GWAS sample size. This is mainly explained by two reasons. Firstly, there is limited evidence that epistasis between the HLA and non-HLA genes, as well as between two non-HLA genes comprise a large fraction of total genetic variation in autoimmune diseases. Secondly, in the additive effect model, the loss of information between genotyped SNPs and causal variants is proportional to linkage disequilibrium (*r*^2^), but in epistasis, the loss of information between the two is proportional to *r*^4^ [80]. Therefore, a much larger sample size is necessary to detect epistasis than to detect the main effect in GWAS [81].

## 7. Epigenetic Regulation

**Figure 2:**
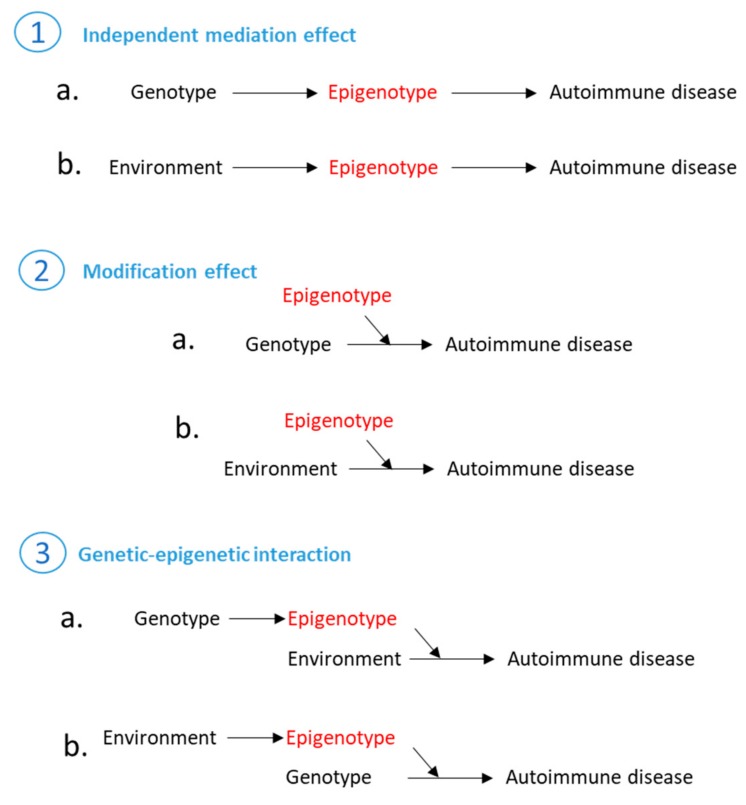
Schematic representation of three modes of actions where epigenetic regulations can take place to contribute to autoimmune disease risk. Figure was adapted and modified from [91].

Epigenetic mechanism is another layer of regulation that influences a gene function. Although the Greek prefix ‘epi’ indicates an effect that is acting ‘on’ the genome, epigenotype can influence autoimmune susceptibility in several ways, as illustrated in Figure 2. As shown in the first scenario in Figure 2, epigenetic mechanisms can independently mediate genetic and environmental risk, which subsequently lead to autoimmune disease. Perhaps the most well-studied epigenetic mechanisms involve DNA methylation, histone modification, and long non-coding RNAs. DNA methylation occurs at the cytosine-phosphate-guanine (CpG) residues; it has been shown that approximately 20% of DNA methylation variance is explained by additive genetic variance [82]. Single nucleotide polymorphisms that are in close proximity to the CpG site (cis, <1 Mb centred on the SNP) as well as SNPs that are far away from the CpG site (trans, >1 Mb centred on the SNP) can both influence DNA methylation levels [82,83]. These SNPs are known as methylation quantitative trait loci (mQTLs). Many early studies attempted to find mQTL associated DNA methylation changes by performing epigenome-wide association analyses [84,85,86]. However, one needs to carefully interpret these results, because it is difficult to discriminate causality (that DNA methylation is causally influencing a trait) from reverse causation (that DNA methylation is a consequence of changes in gene expression), linkage disequilibrium confounding (that causal variants and mQTLs are simply in linkage disequilibrium) or from horizontal pleiotropy (that mQTLs alter DNA methylation and autoimmune diseases via different mechanisms) [87]. Recent developments in statistical strategies such as causal inference test [88], Mendelian randomisation (MR) [89,90], and genetic co-localisation fine mapping [87] have made such discriminations possible.

Liu and colleagues applied a causal inference test on a cohort of 354 rheumatoid arthritis patients and 337 controls to investigate whether genetic risk of rheumatoid arthritis is mediated by DNA methylation. They identified a large number of differentially methylated CpG sites within the HLA region (535 SNP-CpG pairs) that potentially exhibit mediatory effect. A similar observation was made at a non-HLA locus, within the Glutathione S-Transferase Alpha 2 (*GSTA2*) gene [92]. Other studies using the same approach have found that DNA methylation potentially mediate the expression of a number of genes in human islets, including *HLA-DQB1*, the main predisposing gene to type 1 diabetes [93]. In our recent work, we applied Mendelian Randomisation together with genetic co-localisation fine-mapping to study whether DNA methylation mediates the genetic risk of type 1 diabetes. We identified a number of loci, including *CTSH*, *PTPN2* and *AFF3*, where DNA methylation is potentially on the causal pathway to type 1 diabetes [94]. Richardson et al. further extended this statistical framework to systematically investigate the functional roles of DNA methylation in hundreds of traits including many other autoimmune diseases [95]. DNA methylation appeared to increase autoimmune disease risk in a number of susceptible loci, where inflammatory bowel disease had the greatest number of DNA methylation mediated loci, followed by rheumatoid arthritis and Crohn’s disease [96].

DNA methylation may also play a role in the female predominance observed in autoimmune diseases [97]. Part of the reason for the female predominance was thought to be due to a high frequency of skewed X-chromosome inactivation. In females, one copy of the X-chromosome was silenced by DNA methylation whereas the expressed copy of X-chromosome was unmethylated. The choice of which copy of X-chromosome to be silenced is random, which results in two distinct cell populations, where 50% paternal X-chromosome genes are expressed in one cell population and 50% maternal X-chromosome genes are expressed in another. As a result, the dosage of proteins transcribed from X-chromosomes is approximately equal between males (XY) and females (XY). However, not all women have a 50:50 ratio of cells with one or the other X-chromosomes active. A deviation from equal inactivation of each parental allele is known as skewing; for example, some alleles could be inactivated in 70–80% cells and in extreme cases, in 90–95% cells. This may lead to an altered dosage of proteins to be translated in some females. In peripheral blood mononuclear cells and multiple other tissues, skewed X-chromosome inactivation has been observed more frequently in female patients with autoimmune diseases [98,99]. It is considered that lowered expression of X-linked self-tolerance genes in the thymus may lead to lack of exposure of self-antigens, subsequently leading to the escape of autoreactive T cells.

In the second scenario, an epigenotype is thought to modulate a genetic/environmental risk factor. A typical example of this is the regulation by long non-coding RNAs (lncRNAs). Long non-coding RNAs are RNAs that exceed 200 nucleotides in length and they are broadly classified into five subclasses: stand-alone lncRNAs (or large intergenic non-coding RNAs, lincRNAs), natural antisense transcripts (NATs), pseudogenes-derived lncRNAs [100], long-intronic lncRNAs, and promoter/enhancer-associated lncRNAs [101]. lncRNAs have been shown to involve in anti-viral responses [102], T cell differentiation [103], and NFkB signalling [104]. Mirza et al. overlapped known lncRNAs with susceptible variants for inflammatory bowel disease and type 1 diabetes based on their physical locations in the genome. They identified over 2000 inflammatory bowel disease-associated SNPs physically located within 468 lncRNAs and over 1000 type 1 diabetes SNPs within 247 lncRNAs; many of them potentially disrupt the secondary structure of lncRNAs [105]. The authors therefore hypothesised that some of the autoimmune disease associated SNPs can alter the expression and function of lncRNAs, which subsequently influence disease related genes. This hypothesis was systematically investigated using statistical approaches by Kumar et al. where the authors found that disease associated expression quantitative trait loci (eQTLs) affect 112 out of 2140 lncRNAs in whole blood [106]. Hrdlickova et al. mapped lncRNAs expressed in seven immune cell types (granulocytes, monocytes, NK cells, B cells, memory T cells and naïve CD8+ cells) to susceptible loci in nine autoimmune diseases [107]. They found that the proportion of lncRNAs expressed in autoimmune disease loci were significantly higher than the proportion of lncRNAs expressed genome-wide; additionally, the expression levels of lncRNAs in autoimmune disease loci were higher than that detected at the genome-wide level [107]. More interestingly, lncRNAs overlapping the disease regions tend to be tissue-specific. For example, inflammatory bowel disease associated lncRNAs are preferentially expressed in NK cells, juvenile idiopathic arthritis associated lncRNAs are enriched in memory and CD8+ T cells [107].

The third scenario elucidates the most complicated situation, which is genetic-epigenetic interaction. This type of interaction has been seen in many occasions involving enhancer regulations. Enhancers are defined as cis-acting DNA sequences that can increase the transcription of genes. Ninety eight percentage of enhancers are located in the non-coding regions of the genome, either upstream or downstream of genes, or in introns. Enhancers can be identified using high-throughput sequencing targeting specific markers such as H3 acetylated at lysine 27 (H3K27ac) and H3 monomethylated at K4 (H3K4me1), which are chemical modifications of the histone proteins that wrap around DNA. Their activities can also be specific to a tissue or a particular cell type, a time-point in life, or a unique physiological state [108]. A recent study overlapped causal variants with histone marks in 21 common autoimmune diseases showed that causal variants of a disease trait are enriched in enhancers specific to disease-affecting tissues. For example, causal variants of Alzheimer’s disease are enriched in enhancers in the brain; causal variants of type 1 diabetes are enriched in enhancers in lymphocytes as well as in pancreatic islets [109]. In addition, a disproportionate number of enhancers respond to ex vivo stimulation, reflected by increased H3K27Ac (marks active promoter and enhancer) signals and non-coding RNA transcription upon immune cell activation [109].

A specific example of genetic-epigenetic interaction was previously described in Grave’s disease. One hypothesis for the initiation of Grave’s disease is viral infection. Infection can lead to the recognition of auto-antigens via molecular mimicry, which further causes bystander activation of auto-reactive T cells and global pro-inflammatory cytokine production [110,111,112]. To mimic the consequence of viral infection, Stefan et al. treated human thyroid cells with pro-inflammatory cytokines [113]. They observed that thyroid cells exerted significant changes of H3K4me1 signatures at the intron1 of *TSHR*, which harbours a previously predicted causal variant, rs12101261, to Grave’s disease. After pro-inflammatory cytokine treatment, rs12101261 was able to interact with histone deacetylase and a transcription repressor PLZF, resulting in reduced *TSHR* expression and breakdown of central tolerance [113].

An immediate problem of studying genetic-epigenetic interaction is that only 10–20% causal variants were predicted to disrupt transcription factor binding motifs at the enhancer sites, 80–90% causal variants function by modifying the non-classical regulatory sequence [109,114]. In addition, the nature of stimulus-dependent enhancer interactions makes it challenging to robustly study them in un-stimulated cells. The advances in Clustered Regularly Interspaced Short Palindromic Repeats (CRISPR)-Cas9 technologies enabled their screening and characterisation more readily in un-stimulated cells. CRISPR activation (CRISPRa) utilises guide RNAs conjugated with a strong transcriptional activator (i.e., VP64) to induce the expression of endogenously weakly expressed genes [115]. Simeonov et al. recently used this approach to scan enhancers surrounding the *CD69* and *IL2RA* [116]. They identified a CRISPR responsive enhancer at the intronic region of *IL2RA*, and confirmed the ability of the candidate causal variant rs61839660 (group A SNP) to disrupt this enhancer activity in a stimulus dependent manner. Using mouse models, they subsequently showed that upon T cell stimulation, this enhancer controls CD4 naïve T cell polarisation, as CD4+ naïve T cells in the enhancer deletion mouse strain tend to favour a pro-inflammatory Th17 cell differentiation rather than T regulatory cell differentiation [116]. Interestingly, enhancer disruption of *IL2RA* delayed its expression, which was eventually recovered three days after T cell stimulation [116], implying that the induction of autoimmunity could happen in a transient and tissue specific manner.

## 8. Conclusions

With GWAS studies, we have gained significant knowledge to broadly define autoimmune disease-associated regions genome-wide. Although GWAS have their inherent limitations, it was a big step forward, considering that the concept of autoimmunity was initially proposed during the 1940s [117] and the HLA associations were only first described in the 1970s. The challenge for the next decade is to precisely characterise the functions of disease risk loci. With fast-growing profiling of the genomic and epigenomic regulatory elements, as well as cutting edge bioinformatic and molecular genetic tools available, we are in a unique position to study complex loci. Only by understanding the complex and dynamic nature of autoimmune disease mechanisms, can we deliver truly translational research that impact future clinical care.
